# Seismic monitoring in the oceans by autonomous floats

**DOI:** 10.1038/ncomms9027

**Published:** 2015-08-20

**Authors:** Alexey Sukhovich, Sébastien Bonnieux, Yann Hello, Jean-Olivier Irisson, Frederik J. Simons, Guust Nolet

**Affiliations:** 1 UMR 6538 Domaines Océaniques, Université Européenne de Bretagne, Université de Bretagne Occidentale, CNRS, IUEM, 29280 Plouzané, France; 2 Géoazur, Université de Nice, UMR 7329, 06560 Valbonne, France; 3 Sorbonne Universités, UPMC Univ Paris 06, CNRS, Laboratoire d'Océanographie de Villefranche (LOV), 06230 Villefranche-sur-Mer, France; 4 Department of Geosciences, Princeton University, Princeton, New Jersey 08544, USA

## Abstract

Our understanding of the internal dynamics of the Earth is largely based on images of seismic velocity variations in the mantle obtained with global tomography. However, our ability to image the mantle is severely hampered by a lack of seismic data collected in marine areas. Here we report observations made under different noise conditions (in the Mediterranean Sea, the Indian and Pacific Oceans) by a submarine floating seismograph, and show that such floats are able to fill the oceanic data gap. Depending on the ambient noise level, the floats can record between 35 and 63% of distant earthquakes with a moment magnitude *M*≥6.5. Even magnitudes <6.0 can be successfully observed under favourable noise conditions. The serendipitous recording of an earthquake swarm near the Indian Ocean triple junction enabled us to establish a threshold magnitude between 2.7 and 3.4 for local earthquakes in the noisiest of the three environments.

## Abstract (web summary)

Our understanding of the internal dynamics of the Earth is limited by the lack of seismic data available from oceanic domains. Here, the authors use observations from floating submarine seismographs to show that this technique may provide seismic data to fill the gaps in our knowledge.

## Graphical abstract

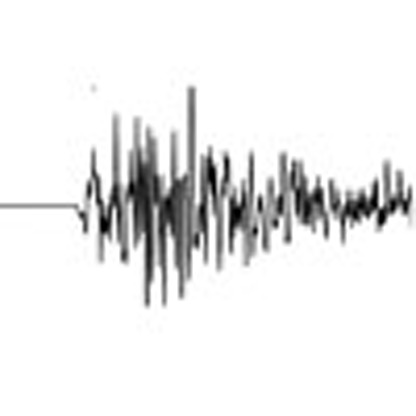

Seismic monitoring in the oceans has been limited to rather localized experiments, due to the high cost of deployment and recovery of instruments such as ocean bottom seismometers (OBSs)^1^,^2^ and moored hydrophones^3^,^4^. Considering that about 70% of the Earth is covered by water, advances in seismic monitoring and imaging of the Earth's mantle via seismic tomography depend critically on the development of radically new instruments, capable of providing seismic data at significantly lower cost. Simons *et al.*^5^,^6^ proposed such a new instrument, known as MERMAID (Mobile Earthquake Recording in Marine Areas by Independent Divers), that is a submarine buoyancy-matched float equipped with a hydrophone to detect acoustic signals generated when seismic waves from the Earth's interior refract into the water column at the sea bottom. In early tests^6^ MERMAID prototypes were passive continuously recording floats that needed to be recuperated after a few days to recover the data. In this work we report on the final development of an autonomously functioning MERMAID^7^, capable of recognizing earthquake waves and transmitting seismograms in quasi-real time.

## Results

### MERMAID design and operational principles

The MERMAID uses a popular Autonomous Profiling Explorer float (Teledyne Webb Research), also employed by oceanographers in the Argo^8^ program. The float is equipped with a Rafos II (Benthos) hydrophone sensitive down to 0.1 Hz, dedicated electronics, and software for signal analysis. Signal transmission by Iridium satellite is two way and allows for modification of filtering and detection parameters, as well as of the operating depth (typically between 700 and 2,000 m). The full control over the recording strategy offered by the two-way communication makes the MERMAID a highly flexible recording instrument and contrasts it drastically with the OBSs and moored hydrophones. The MERMAID drifts passively, typically with a speed of several km per day, until an earthquake signal is detected. If this is identified as a strong P wave, the MERMAID ascends for transmission of the recorded waveform as well as its global positioning system (GPS) coordinates at the surface. P waves of smaller amplitudes and/or with a less certain identification are stored in a buffer for later transmission (for these we must interpolate to estimate the float's position at the time of recording). Detection and identification of seismic and other signals is done using a discrimination algorithm^9^ specially developed for this purpose (see Methods).

Small anomalies (with respect to predictions made by using radially varying Earth models) in observed onset times of P waves are indicative of aspherical temperature or compositional variations in the Earth's mantle along the ray path. If there are many delays along different paths, they can be imaged^10^. A significant advantage of MERMAIDs is their mobility, because this enables them to cover large areas without duplicating information (for example, time delays along the same ray paths for aftershocks). The relatively low cost of a float, combined with data transmission in quasi-real time, makes expensive recuperation operations unnecessary, and the floats, powered by batteries, are generally lost at the end of the battery life (as are the oceanographic Argo floats). While current MERMAIDs carry enough battery load to function for about 2 years, the second generation of MERMAIDs (currently under development) will have a lifetime of 6–8 years with lithium-ion batteries, and perhaps three times that much in the future (if lithium–sulfur batteries fulfil their promise). This makes it likely that floats will sooner or later approach land close enough to be recovered and redeployed after battery replacement, which is important from both economical and environmental points of view.

### Detection of P waves

After extensive testing in shallow water, the first two autonomous MERMAIDs were launched in December 2012 in the Ligurian Sea (Mediterranean). One was recovered after 10 months when it entered an area with heavy fishing activity, north of Barcelona (Spain). The second was recovered after 18 months near Málaga (Spain), when the state of its battery would not have permitted more than 30 additional surfacings. These deployments served to test hardware, software and the performance of the discrimination algorithm under various noise conditions, which are strongly related to weather. After these pilot deployments, more MERMAIDs were launched; in total, three MERMAIDs are currently operating in the Mediterranean Sea for the purpose of seismic P-wave tomography of the mantle under this complex oceanic basin.

During 2 years (from January 2013 to December 2014) the MERMAIDs in the Mediterranean Sea recorded more events than initially anticipated. For the purpose of quantification we define the number of potential seismograms as the number of seismic signals from earthquakes with a moment magnitude *M*=6.5 or higher, arriving at MERMAIDs within an epicentral distance of 100°, the range in which mantle P waves arrive. The ratio between the number of seismograms transmitted and the potential number is our success rate. For the specified period, the success rate is 51% (57 P waves). Even with the lower threshold magnitude of 6, the success rate still remains acceptable (27%). In total, 202 seismograms were transmitted, among which 65 seismograms of even smaller-magnitude earthquakes, and 43 of the events with epicentral distances exceeding 100° (PKP waves traversing the core).

Deployment in the Southern Indian Ocean began in March 2013 with two MERMAIDs launched north of Amsterdam and St Paul Islands to test the performance under much noisier conditions. Two more launches occurred in November 2013, ∼1,400 km east of La Réunion Island.

**Figure 1:**
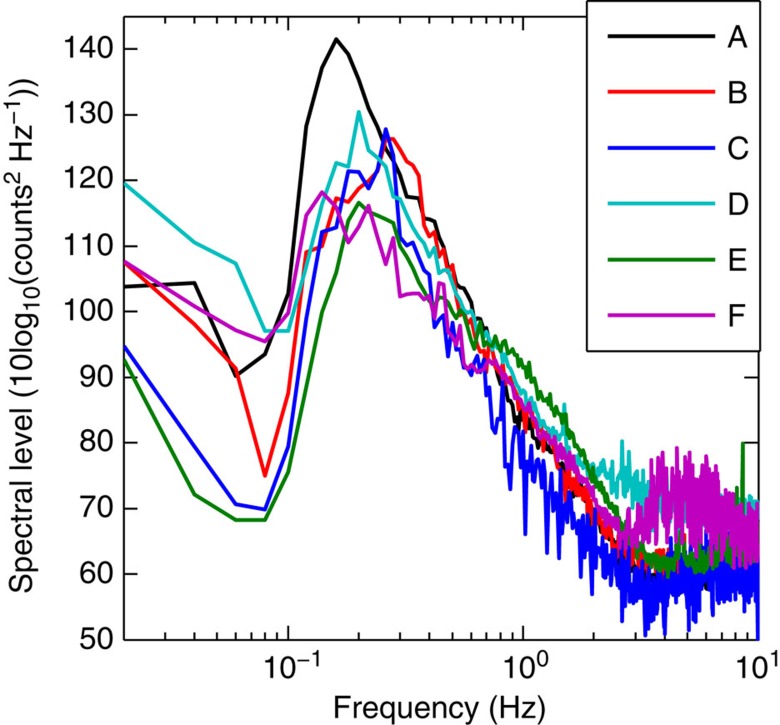
Power spectral density of the ocean ambient noise estimated from the data sent by MERMAIDs. Each curve represents the power spectral density estimated from the data sent by one MERMAID. For each curve, we specify the area, the average location and the deployment depth of the MERMAID during the data acquisition. The Indian Ocean: A (31.5° S, 80.2° E, 2,000 m), B (37.2° S, 70.5° E, 1,500 m), C (24.7° S, 69.7° E, 2,000 m) and D (21.6° S, 69.5° E, 1,500 m); the Mediterranean Sea: E (43.5° N, 07.9° E, 1,500 m); the Pacific Ocean: F (03.5° S, 93.0° W, 1,500 m).

The average ambient noise in the Indian Ocean differs significantly from that in the Mediterranean Sea (Fig. 1), showing high spectral power at lower frequencies (<1 Hz). Noise samples obtained from false triggers were used to find the necessary adaptations to the discrimination algorithm. The filter in the trigger algorithm was set to a high-pass corner frequency near 1 Hz, which eliminates most of the low-frequency ambient noise. This significantly increased the number of detected P waves.

**Figure 2:**
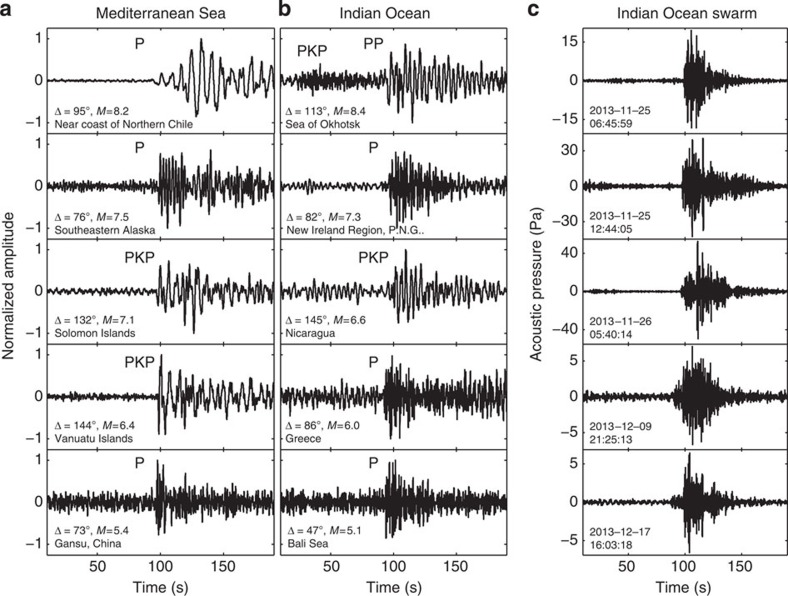
Representative seismograms transmitted by MERMAIDs. (**a**) Teleseismic events detected in the Mediterranean Sea. The amplitudes of all seismograms are normalized to one. Each panel specifies a seismic phase, an angular distance Δ between MERMAID and an event's hypocenter, magnitude and region. (**b**) Idem, in the Southern Indian Ocean. The Nicaragua signal was high-pass filtered at 0.1 Hz, whereas the last two seismograms were filtered with the pre-STA/LTA filter to amplify the P-wave onset. (**c**) Low-magnitude underwater earthquakes detected by the MERMAID during the swarm in the Indian Ocean. Each panel indicates the UTC time of the signal's trigger. All signals are filtered with the pre-STA/LTA filter described in the text. Amplitude scale (originally in counts) is scaled to represent the pressure in Pa at 2 Hz, which is the dominant frequency for the detected signals.

We present the Indian Ocean statistics for the 1-year period (since January 2014, the moment when optimal detection parameters were implemented, to December 2014). Currently, the success rates are 35% (50 P waves) and 17% for the events with magnitudes *M*≥6.5 and *M*≥6.0, respectively. The total number of transmitted seismograms is 104, including 23 seismograms of events with *M*<6.0 and 9 of those occurring at distances larger than 100°. Figure 2 shows some of these seismograms.

In May 2014, 10 MERMAIDs were launched in the Pacific Ocean to study the deep structure of the Galápagos mantle plume, which is suspected to arise from the top of the lower mantle^11^. For the period from June 2014 to December 2014 the statistics of the deployment in the Pacific show a success rate even superior to that in the Mediterranean: 63% (75 P waves) and 30% for the events with magnitudes *M*≥6.5 and *M*≥6.0, respectively. A total of 236 seismograms were transmitted, including 90 seismograms of the events with *M*<6.0 and 40 of the earthquakes located at distances larger than 100°.

In summary, the success rates in all three oceanic basins can be presented as follows: a float will record a P or PKP seismogram on average every 7–8 days in the Mediterranean Sea or in the Pacific Ocean and every 14 days in the Indian Ocean.

### Earthquake swarm observation

**Figure 3:**
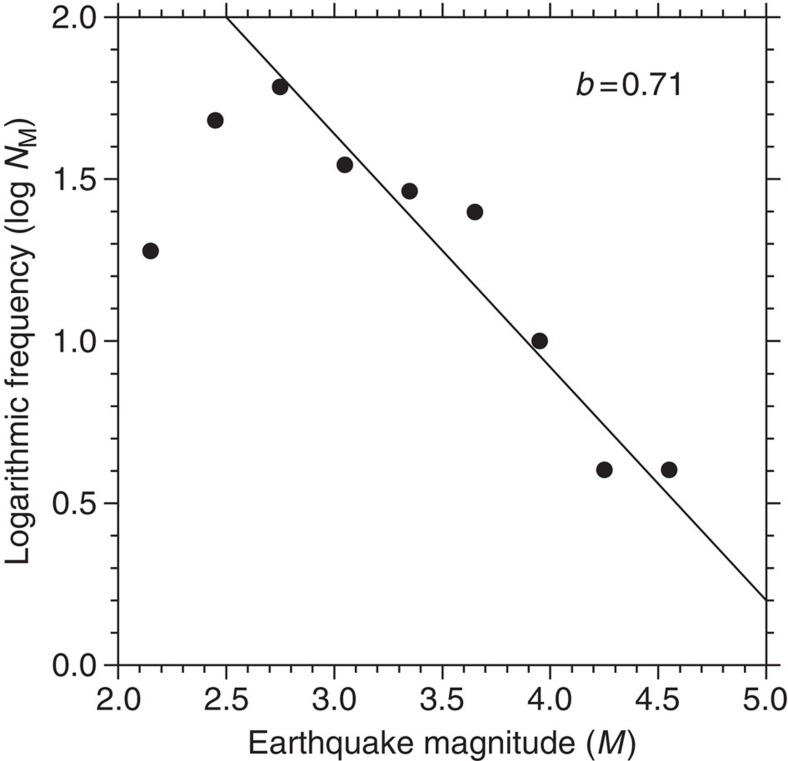
Gutenberg–Richter plot of the swarm events detected by one of the MERMAIDs in the Indian Ocean. Logarithmic relation between the number *N*_*M*_ of swarm earthquakes with magnitude *M*. The slope of the graph (the *b* value) is indicated on the plot.

In November-December 2013, one of the floats in the Indian Ocean transmitted 235 seismograms from a large earthquake swarm (that is, sequences of many earthquakes occurring in a relatively short period of time) near the triple junction at 70° E, 25° S where three ocean spreading ridges come together and where the African, Indian and Antarctic plates meet (Fig. 2). At the same time, land stations recorded only 25 of these events, none with a magnitude smaller than 4.4 and all but two within 4 days of the main shock (*M*=5.1) on 24 November 2013 (because of buffer overflow the MERMAID missed all but one of these stronger events). Only the limitations in the memory buffer size prevented detecting many more small-magnitude earthquakes that must have occurred during the swarm; even with some gaps in the recording, the analysis of the detected events shows that the activity continued until 23 December 2013 when it stopped rather abruptly. This clearly demonstrates that MERMAIDs can also detect small-magnitude events that are never recorded on land. Comparison of a large-magnitude event that was also recorded on land allowed us to estimate magnitudes for other recorded events and to construct the Gutenberg–Richter plot of log*N*_*M*_ versus magnitude, where *N*_*M*_ is the number of events of magnitude *M*. The relationship is linear with an apparent *b* value (slope) of 0.71 down to magnitude 2.7 (Fig. 3), which might indicate that few or no events of magnitude 2.7 would have been missed if the buffer had allowed for them to be stored for later transmission. However, this value for *b* is rather low^4^—if we constrain *b* to have a more common value near 1.0, a new threshold magnitude of around 3.4 is obtained. The smallest event recorded had a magnitude as low as 2. The serendipitous recording of this swarm also allowed us to assign an upper limit of 500 m for the difference between the GPS location at the time of surfacing and the location at the time of recording (during the entire duration of the swarm the deployment depth was 1,500 m), which gives a very small error in the tomographic interpretation of onset times (see Methods).

## Discussion

**Figure 4:**
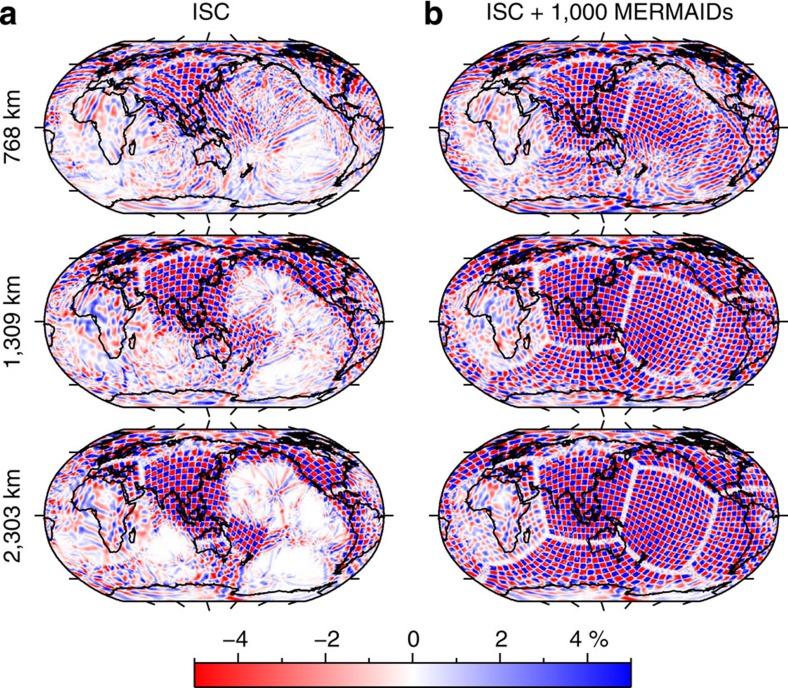
Resolution test. At every depth, the Earth is filled with six chequerboard patterns. Each pattern is composed of regions in which P-wave velocity is by 5% either higher (blue colour) or lower (red colour) than the P-wave velocity given by the reference model IASP91 (ref. 21). The anomalies' lateral size varies from about 200 km in the lower mantle to 300 km at 768-km depth. Comparison of the patterns obtained when using (**a**) only the data provided by the ISC stations and (**b**) combined data of the ISC stations and the MERMAIDs shows a significant improvement of the resolution in the ocean basins at all depths.

We conclude that we have been able to routinely record large distant seismic events under very different noise conditions. This success opens up the world's oceans for seismic P-wave tomography of the deep mantle. To demonstrate this unambiguously, we used the detection statistics already obtained with the currently deployed MERMAIDs to model the expected yield for a network of 300 and 1,000 MERMAIDs, for a period of 5 years (the minimum lifetime for the next generation of such instruments). We then performed a ‘resolution test' 10, in which we test how well a synthetic data set for a known model can be imaged. Figure 4 shows the images of a model, in which the six sides of a ‘cubed earth' parametrization^12^ were filled with a chequerboard pattern. Using only delay times reported by the International Seismological Center (ISC) from existing seismic networks, the chequerboard pattern beneath the oceans is not reproduced except for a few small areas. Adding 5 years of data expected from an array of 1,000 MERMAIDs yields good resolution at all depths for most of the area covered by the ocean except for the Southeastern Atlantic, where the synthetic MERMAID coverage is less. Even though each measurement is at a unique location and we added errors with a s.d. of 0.4 s, the least-squares inversion clearly reproduces the anomalies almost to their full input amplitude of 5%.

The results of the first deployments have motivated us to start developing an even more powerful float that has a longer lifetime, and will be able to accommodate up to eight different sensors for multidisciplinary monitoring of the oceans, such as high-frequency biological sounds (marine mammal vocalizations)^13^, meteorological sounds triggered by rainfall^14^ and biogeochemical data. The observation of small earthquakes down to magnitude 2 shows that MERMAIDs could be deployed after an earthquake to locate aftershocks. For this we will have to record continuously into a large buffer and auto-locate a small array of MERMAIDs using acoustic signals between pairs of floats. Our web page (https://www.geoazur.fr/GLOBALSEIS/Data.html) provides an up-to-date access to the data collected by all MERMAIDs as well as the files containing their trajectories.

## Methods

### Detection

The detection of seismic signals is ensured by continuous calculation of the ratio of short-term to long-term moving averages (STA/LTA algorithm)^15^. Each detected signal is then analysed by a dedicated discrimination algorithm^9^ that relies on signal processing using the wavelet transform. For the purpose of seismic P-wave tomography MERMAIDs particularly target teleseismic (that is, generated by distant earthquakes) P waves.

After initial experiments in the Pacific Ocean^6^, the Mediterranean Sea served as a testing ground during the development of MERMAID's hardware. Since the discrimination algorithm was designed using data collected in the same area, it was particularly well adjusted for the discrimination of teleseismic P waves in the ambient noise conditions of the Mediterranean Sea. Though we are unable to reprogram at distance the discrimination algorithm stored in the MERMAID-processing board, two-way communication via Iridium allows us to manipulate digital filters and adapt acceptance parameters in the algorithm (for the satellite communication, we are using the RUDICS service that allows the transmission of large amounts of data at a reasonable cost). This allowed us to improve the detection of seismic signals in the Indian Ocean after first noise samples were obtained. We can also adapt the length of the seismogram and the degree of its compression during transmission. The data presented here were sampled at 40 Hz, but transmitted (after wavelet compression) at either 20 or 5 Hz. The pre- and post-trigger windows are adaptable, but generally have a length of 100 s each, giving 200-s long seismograms. During initial tests with longer windows, we have not observed any arrivals that could reliably be identified as teleseismic shear waves converted into acoustic waves at the ocean bottom.

### Adaptations for the Indian Ocean

MERMAID's software allows numerical filtering of the recorded signal before running the STA/LTA algorithm (even though the transmitted seismograms remain unfiltered). To counter the effect of much higher noise at low frequencies, the high-pass corner frequency of this filter was raised from 0.1 to 1.0 Hz. This resulted in fewer false triggers, though the ability to discriminate between T waves (the waves caught in the sound fixing and ranging channel and thus travelling mostly in the water) and P waves was affected. The efficiency of the discrimination algorithm was restored by modifying its parameters. The experience in the Indian Ocean showed us that it is extremely important to have a discrimination algorithm adaptable to different local noise conditions, since a judicious choice of parameters leads to a smaller fraction of missed P waves or false triggers. MERMAIDs in the Pacific Ocean were launched with the same filter settings as those in the Indian Ocean. These filter settings seem to work well, though it is too early to judge whether this will hold true for a full seasonal cycle.

### Swarm analysis

One MERMAID launched near the Indian Ocean triple junction detected an earthquake swarm, which began with a small foreshock immediately after the launch on 24 November 2013, at 04:08 UTC, followed by the main event of magnitude 5.1 on 24 November 2013, at 22:04 UTC, which was observed by land stations and reported by the National Earthquake Information Center (NEIC). The MERMAID that, for the first dive, had been programmed to surface after 24 h to transmit its state-of-health parameters, recorded the foreshock; the main event was not recorded since it happened while the MERMAID was at the surface.

The first seismogram transmitted on 25 November 2013 has a time stamp of 03:58 UTC, 6 h after the main shock. The MERMAID missed all but one of the eight aftershocks listed by the NEIC catalogue for the period directly following the main shock up to the time that the buffer, which can contain up to 8 Mb (or at most 128 seismograms), became full. This happened on 26 November 2013, at 06:42 UTC, as deduced from the time stamp of the last recorded seismogram since the start of the sequence. The next surfacing did not occur until 2 December 2013. At this moment we programmed the MERMAID to surface frequently so that by the end of the swarm the MERMAID had surfaced 11 times. In total, 235 signals generated during the swarm by low-magnitude earthquakes were transmitted, all but one too weak to be detected by any land station.

According to the NEIC catalogue, this event had a magnitude of 4.6 and occurred on 25 November 2013, at 19:36:59 UTC. The calculated distance between the event's epicentre (25.44° S, 70.03° E) and the MERMAID's surfacing location was only 11 km, which is most likely within the uncertainty ellipse that encloses the epicentre. A comparison of the magnitude for this earthquake with the observed peak-to-peak amplitude of the pressure signal allowed us to assign a rough magnitude to all other swarm earthquakes (neglecting possible variations in epicentral distance), and to obtain valuable information about the ability of MERMAIDs to record microseisms. Applying a high-pass filter with a corner frequency at 1 Hz, we established an empirical amplitude–magnitude relationship *M*=log_10_*A*−3.38, where *A* is the amplitude in counts (in the high-frequency limit 8.3 × 10^4^ counts equal 1 Pa)^16^.

### Drift calculations

Since the exact location of the MERMAID at the time of a P-wave recording is not known, we adopt the location obtained by the GPS localization immediately after surfacing. This implies that the horizontal drift during an ascent might be a source of error in seismic tomography performed from the data acquired by MERMAIDs and it is important to estimate it (the discussion below does not apply to the events stored in the memory; for these events interpolation should be used, which may lead to larger errors). This drift is due to the wind-driven near-surface currents that are much stronger than the currents at depth. The direction of the near-surface current can also be significantly different from that of the current at depth. Ideally, one would like the MERMAID to dive to its cruising depth and come up immediately, which would give a direct measurement of the horizontal drift during diving and surfacing, combined. Unfortunately, this is not possible with the existing float, but several short consecutive dives during the swarm recording allow us to estimate an upper limit for the amount of drift experienced by the MERMAIDs while ascending. Once we had observed the first swarm data, the MERMAID was allowed to return to the surface more often, which resulted in frequent surfacings (on 2, 3, 5 and 6 December 2013) separated by relatively short trajectories at depth. We designate with *D* the known distance between the last GPS location recorded just before diving and the first one immediately after surfacing. *D* is the vectorial sum of three drifts: the one while diving, which is mostly concentrated in the top 100 m and dominated by wind (**A**); the slow drift at the programmed depth, while passively cruising with the deep ocean current (**B**); and the drift in the upper layer when coming up (**C**). Therefore, *D*=|**A**+**B**+**C**|. We are interested in *C*=|**C**|, since this is the difference between the location reported by GPS and the recording location. It should be noted that the MERMAID rises much faster (in about 1.5 h) than it dives (6 h), thus |**A**|≫|**C**|. It is not possible to separate the three factors (the direction of the deep drift varies slowly, whereas the surface drift may change with the wind), but it is clear that the upcoming passage through the upper layer, and thus the location error *C*=|**C**|, must on average be much smaller than *D*. We recorded several *D*s for short dives: 1.12 km in 32 h (3 December), 1.80 km in 33 h (6 December) and 2.67 km after a 91-h long dive (10 December). If we assume for convenience that all drifts are in the same direction, then the difference between the last *D* with the first one indicates a deep drift *B*=|**B**| of 2.67−1.12=1.55 km in 91−32=59 h, which in case of the dive on 3 December translates into 1.55 × 32/59=0.84-km deep drift, and, which leaves 1.12−0.84=0.28 km for |**A**+**C**|. Setting **A**=4**C** for a total near-surface drift of 5**C** gives *C*=0.28/5=0.056 km. If instead we assume that the surface flow is exactly in the opposite direction of the deep flow, then *C*=(1.12+0.84)/5=0.39 km. For the second dive we find in the same way 0.26 and 0.53 km. Though these calculations are order of magnitude at best, and only for the surface drift in the Indian Ocean and under a limited range of wind conditions, these estimates correlate well with our much more general experience of the retrieval of OBSs, for which the drift can be measured precisely since their positions at the sea bottom are known. The OBSs are rarely found more than 100 m from the point of launch, even though they traverse the full water column (which can be several km deep) as they rise. The location error due to the drift is of no importance in seismic tomography. A typical teleseismic P wave has a horizontal phase velocity of 20 km s^−1^, which implies that a drift error of 0.5 km gives a timing error of 0.025 s, well below the estimated error in the reading of teleseismic onset times.

### Resolution test

**Figure 5:**
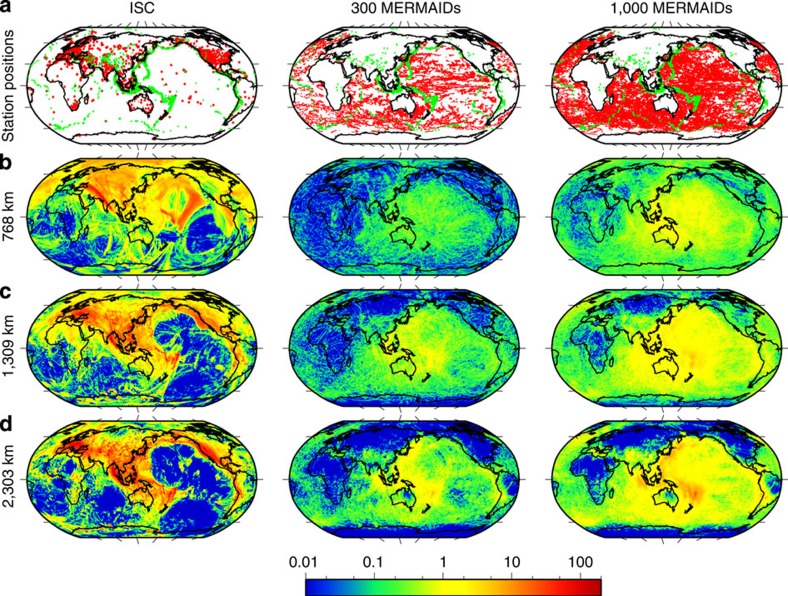
Ray coverage. (**a**) Red dots indicate both positions of the land stations, whose records comprise the ISC data base, and the simulated positions of the MERMAIDs. Note a virtually uniform distribution of the MERMAIDs thanks to their mobility. Green dots indicate epicentres of the earthquakes listed in the ISC catalogue for the period from 2004 to 2008. (**b**–**d**) The 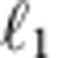-norm of columns of the tomographic matrix. For any column, its 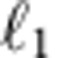-norm is proportional to the total length of rays crossing the volume cell corresponding to that column. Colour scale is in arbitrary units.

To model MERMAID trajectories realistically, we used trajectories of existing Argo floats recorded over a 5-year period (2004–2008). We assigned probabilities for the observation of a P-wave delay time from a teleseismic event in agreement with the observations reported here (from only 10% for 5.5<*M*<6 to 100% for *M*>7). For nearby events we assumed that all events of magnitude *M*>5.5 and *M*>6 are recorded if they occur within a distance of <15° and between 15° and 30°, respectively. These regional events have their ray paths mostly in the upper mantle and contribute little to lower-mantle resolution. Comparison with the ISC earthquake catalogue gives 102,080 and 341,607 delay times expected to be recorded by a network of 300 and 1,000 MERMAIDs, respectively. To estimate the parallel output of the land-based stations, we used the ISC database^17^ of delay times over the same period; as a result, 1,567,829 observed seismic delay times were obtained (only events of a magnitude of 5 and higher were used with conventional selection criteria to assure data quality). Although this number is significantly larger than the number of events expected from the MERMAID network (a longer time period for ISC data would have resulted in even more data but would not fill the gaps in the oceans), the ray coverage in the oceanic domain depends very strongly on the MERMAID presence (Fig. 5). The ray coverage, which is equal or close to zero for much of the oceanic part of the Southern hemisphere when only delay times provided by land stations are considered, is significantly filled in even with 300 MERMAIDs, though one would need 1,000 floats to have a more equal balance with data from land-based stations. The resolution tests in Fig. 4 were carried out with the ray coverage shown in the left and right columns of the Fig. 5. The recovered models satisfy these data with a root mean-squared misfit of one s.e. Errors with a s.d. of 1 s were added to the ISC data^18^; MERMAID onset times so far have been hand-picked, and our initial tests on the waveforms recorded in the Ligurian Sea point to an average accuracy of 0.4 s, which is compatible with that from nearby land stations. This error was used in the modelling but we expect it to be reduced further by adapting wavelet-based techniques^19^ for picking P-wave arrivals under observed ocean noise conditions. In general, because reverberations in the water layer have a dominant influence on the recorded waveforms, waveform correlations can only be done for nearby MERMAIDs and thus manual determination of the onset times is preferred over more sophisticated cross-correlation methods.

Other potential sources of error for the tomographic images arise from the need to correct onset times for the time P wave travels in the water column. The determination of this correction is influenced by the sound velocity variability and the accuracy with which the local bathymetry is known. The sound velocity in the ocean varies with temperature and salinity, but by <1% and only in the top 500-m deep layer, below which it remains fairly constant^20^. The uncertainty introduced by the sound speed variation within the depth range covered by MERMAIDs (whose average operating depth is 1,500 m) is therefore negligible as compared with the estimated precision of 0.4 s of picked onset times. Corrections for bathymetry are more important. We estimate that the bathymetry (averaged over a seismic Fresnel zone with a typical diameter of about 2 km) must be known preferably with about 150 m precision, which would lead to an uncertainty of at most 0.1 s, again much <0.4 s. The depth of the MERMAID itself at the moment of recording is known from an absolute pressure sensor with an excellent precision of about 1 m.

## Additional information

**How to cite this article**: Sukhovich, A. *et al.* Seismic monitoring in the oceans by autonomous floats. *Nat. Commun.* 6:8027 doi: 10.1038/ncomms9027 (2015).
